# Book Reviews

**Published:** 1862-01

**Authors:** 


### OUR LIBRARY TABLE.

*The Roll of the College of Physicians of London.* Compiled from the Annals of the College and from other Authentic Sources. By William Munk, M.D., Fellow of the College, &c. Vol. II. 1701—1800. Longman. 8vo.

In our last number we noticed, in terms of justly-merited approval, the first volume of this useful and agreeable addition to the history of medicine in England. The second, now before us, fully maintains the character of its precursor for accuracy and conciseness. At first we were inclined to wish that Dr. Munk had entered in more detail into the bibliography of the subjects of his memorial,—such, *e.g.*, as in the instance of Alexander Russell, whose "Natural History of Aleppo" was not the sole emanation from his laborious pen. But, considering the extent to which the "Roll" must have stretched, and the various works of reference that may be consulted readily, we think the plan adopted the most judicious. Besides, any possessor of the "Roll," so disposed, can annotate or illustrate his copy; and few books better admit of such minute elaboration and ornature. We have little to add to Dr. Munk's facts; but we may be permitted to record a characteristic anecdote of the virtuous and accomplished Richard Mead, as preserved by Mr. Robert Chambers, in the last volume of his "Domestic Annals of Scotland." Speaking of the witty and enthusiastic Jacobite, Dr. Archibald Pitcairn, he says: "When the learned physician acted as professor at Leyden, he had amongst his pupils two men of great eventual eminence, Herman Boerhaave and Richard Mead, both of whom entertained a high sense of the value of his instructions. A son of Pitcairn having forfeited his life by appearing in the rebellion of 1715, Mead, then in great favour in high places, went to Sir Robert Walpole to plead for the young man's pardon. 'If I have been able,' he said, 'to save your or any other man's life, I owe the power to this young man's father.' The claim was too strong, and put in too antithetic terms, to be resisted."

Of John Rogerson, mentioned at p. 360, the granddaughter and sole representative married William, 9th Lord Rollo of Duncrub.

It is somewhat remarkable how many dissenting ministers have betaken themselves to the study and practice of medicine.

*A Practical Treatise on the use of the Ophthalmoscope.* By J. W. HULKE, F.R.C.S. Large 8vo, pp. 70. 1861. London: J. Churchill.

Mr. Hulke informs us, in his preface, that, having been "repeatedly asked by the students at the Royal London Ophthalmic Hospital for a guide to the ophthalmoscopic examination of the eye," he determined, in the absence of any standard work in English on the subject, to make some necessary additions to his "Jacksonian Prize Essay" for 1859, and to place it before the profession to supply the indicated want.

In doing so, Mr. Hulke has exercised a sound discretion, and has made students of ophthalmology much his debtors. We can only wish that he had endeavoured to make his treatise more elaborate and complete, and that he had discussed the use of the ophthalmoscope as a guide to treatment, as well as to pathology.

The work is divided into three parts, treating respectively of the optics of the ophthalmoscope; of the ophthalmoscopic appearances of the healthy ocular structures; and of the ophthalmoscopic appearances of diseased structures and congenital imperfections. On each of these subjects Mr. Hulke is lucid, but brief almost to the extent of being meagre.

In the first part we find diagrams illustrative of the course of the rays of light, and the formation of the image, in the different methods of examination. And Mr. Hulke supplies a deficiency of former writers, such as Mr. Hogg, Mr. H. Walton, Mr. Wharton Jones, and Mr. Dixon, by describing fully the so-called "direct method," by which alone a minute study of the ocular structures, in contradistinction to a mere general view, is rendered practicable.

In the second and third parts the student or practitioner, who has mastered the difficulty of seeing with the ophthalmoscope at all, will find most valuable help in the interpretation of appearances. Without such help, nothing but very large experience can enable an observer to fix a limit between healthy variations and morbid conditions; and there can be no doubt that the two are frequently confounded.

The work affords evidence, in almost every page, of the recent influx of ophthalmological technicalities from the German; and these technicalities will sometimes be obscure to those who read them for the first time. We observe, moreover, that Mr. Hulke makes frequent use of the word "equator" in his descriptions, and that he means by it an imaginary circle dividing the globe into anterior and posterior hemispheres. This meaning is perhaps justified by analogy, such an equator having its plane at right angles to the "axis" of the globe. It must be remembered, however, that all English writers of repute have hitherto used "equator" to signify an imaginary circle, dividing the globe into superior and inferior hemispheres; and we think Mr. Hulke would have done well to sacrifice analogy to usage, and to avoid confusing the student by the removal of a descriptive landmark.

Such an error as this is, however, of the less importance, as it is impossible for the ophthalmoscope ever to become an ordinary instrument of diagnosis in the hands of the general practitioner. Ten years have now elapsed since its introduction by Helmholtz; and, while Mr. Hulke, in 1861, publishes the first English systematic treatise upon its use, the form of instrument which he recommends for the "direct method" was not to be obtained, a few weeks ago, from any instrument-maker or optician in London. The so-called "ophthalmoscopes" commonly sold are only calculated to disappoint their purchasers.

While, however, the practical difficulties attending the use of the ophthalmoscope must limit the number of those who master it, it is highly desirable for every practitioner to understand the principles of its application, and the nature of the pathological states that it reveals. For this purpose we cordially recommend Mr. Hulke's welcome and valuable treatise; and we hope soon to see a second edition, embracing a greater variety of topics, and dealing with them at greater length.

The work is illustrated by four plates, well calculated to give a general idea of the appearances to those who have not been able to observe them in the eye; but, like all other ophthalmic illustrations, failing to do justice to the exquisite delicacy and beauty of the structures delineated.

*De L'Hygiene Morale de la Folie appliquée dans le grands Asiles d'aliénés.* Reponse à M. le Docteur Lisle par le Docteur A. Pain, Médecin adjoint de l'asile d'aliénés de Clermont (Oise); ancien interne de Bicêtre et des Hôpitaux de Paris. Paris, 1861: Baillière et Fils, p. 16.

Dr. Lisle having ventured, in a series of letters in *l'Union Médicale*, Tome IX., to bemoan the abandonment of the moral treatment of the insane which he conceives has taken place in France; and, sacrificing the present to the glory of the past, to adduce the diminished number of cures as a proof of his position and the enormous population of modern asylums as an explanation that no other result could be looked for; Dr. A. Pain repels the calumny. He argues first that the crowds of dements found in large asylums are not incurable because they were not treated, or treated imperfectly, but because they were in that state when admitted. He argues, *secondly*, that if due regard be had to calculate the proportion of cures to the number of curable cases received, a larger number will be found to reward the efforts of the medical officers of the large than of small asylums. In Salpetrière, containing 2800 patients, of whom 2005 were regarded as curable, 1233 cures took place in ten years; being in the proportion of 61·49, whereas in Clermont, [Oise] where the gross population amounts to 4257, of whom 1510 were curable, 950 cures took place in the same period, being in the proportion of 62·91. It is perhaps necessary to point out here, that the whole force of such an argument hinges upon the principle pursued in diagnosing the curable in each case, and the accuracy with which it was carried out. He argues, *thirdly*, that it would be unfair to draw any inferences as to our success being inferior to that of Esquirol from such a fact as the rise from 28 per cent. of incurables admitted into Salpetrière from 1804 to 1813; to 81 per cent. in 1853; and that the true test is found in the proportion of cures to that of admissions, which appears to be 25·93 in Salpetrière, and 30·41 in Clermont; although the number of entrants labouring under acute mania and delirium tremens must be much greater in the former than the latter. He argues, *fourthly*, that the noble lessons of Pinel, Esquirol, &c., are no longer slavishly attended to, not because of materialistic tendencies, or the actual state of science, or the overworking of the medical staff, or its imperfect organization in asylums; but in the conviction that it is necessary to combat physical disease, upon which insanity so often depends, physically; on the impossibility of influencing the uneducated insane by means addressed to their understanding; and in the substitution of a moral hygiene which is applied in a spirit of humanity and enlightenment, which places its objects in conformity with the laws of morality, under the empire of a gentle but firm discipline; assimilates their position to that of ordinary life, engages them in occupation, encourages and rewards them; in place of that moral treatment which was applied directly to the eradication of delusions and the subjugation of excitement. Assuredly Dr. Pain is attacking a cloud-spectre of the Hartz mountain, never seen in this country, although we have bugbears and Titanic errors in abundance. He argues, *fifthly*, that in the economy of erection, of maintenance, in the unity of the different services, in the enormous resources for the organization of productive labour, and in the smallness of the public burdens, a large possesses advantages over a small asylum containing 300 or 400 inmates, such as is recommended by M. Girard de Cailleux, the inspector of asylums. He points to Clermont, where three medical men effect a larger number of cures than the ample staff in Salpetrière, and affirms that if provision be made for the separation of the excited from the tranquil, and for concealing, as much as practicable, the indication of compulsory sequestration, all special architectural and other arrangements have been experimentally proved to be unproductive of benefit, if not positively vicious. He *lastly* argues that in place of interfering with time-honoured Bicêtre and Salpetrière, or of multiplying small asylums in the department of the Seine: there should be founded a vast establishment, he does not specify of what size, but possessing extensive grounds, and a system of colonization, which shall prove a prolific source of economy. Naturally and justifiably our author's observations have been coloured by the last recommendation. He is the colleague of M. Labitte,* and writing with the present experience of the benefits of classification and occupation before him, he is not in a favourable position for examining the subject upon its other sides. Without entering upon the vexed questions of colonization, or village asylums, it is, however, incumbent upon us to say that in the case of the colony of St. James, as of somewhat similar establishments, the separate buildings have been regarded as a series of small asylums, and the whole of the arrangements as calculated to relieve the central hospital of a redundant, or superfluous, or embarrassing population; a protestation in fact, against the monster establishment, but an argument in favour of the moral hygiene which he advocates.

*Remarks on Native Education in India, in a Psychological Point of View.* By Dr. S. G. Chuckerbutty, Offg. Professor of Materia Medica and Clinical Medicine, Medical College, Calcutta. 8vo. Calcutta: Kaully & Co. 1861. Pp. 36.

This is a brief, but highly interesting and instructive discussion of the essentials of education among the natives of India, psychologically considered. Simply, but perspicuously and systematically, Dr. Chuckerbutty describes the chief mental and moral phenomena, and in what characteristic manner these are manifested among the natives of India, pointing out how this knowledge guides to sound principles for their education. As an example of the pleasant manner in which Dr. Chuckerbutty has carried out his task, we may quote his remarks on *Imagination.*

---

* *De la Colonie de Fitzjames.* Succursale de l'asile privé de Clermont (Oise) considérée au point de vue de son organisation administrative et médicale, Paris, 1861.

"Imagination is that power of the mind which enables it to conceive objects, qualities and scenes of which it has no real knowledge, and which may or may not exist. Fiction and poetry are pre-eminently works of imagination. This is a power which is very readily and early excited; thus the stories of *Jack the Giant Killer* and the *Dragon of Huntley* have a greater charm for the child than the common details of a plain matter of fact.

"Imagination has its uses as well as its abuses. Exercised upon legitimate affairs, it is a valuable associate to the other mental powers; diverted to sensual and unsubstantial objects, it leads into vice and corruptive influences.

"Thus, when we imagine the feelings of a man on the occurrence of any great catastrophe or success, it is a legitimate use of the imagination, for it promotes sympathy and mutual respect; but when we indulge in fancy on matters of love, that is an abuse of the imagination, because it leads to immorality and disputes.

"To control and guide the imagination becomes hence an object of paramount necessity in the instruction of youth, and to this end must be cautiously directed the attention of parents and guardians in the exercise of the authority they possess over the young.

"Now, to sport in imagination has been heretofore a chief besetting sin of the Indian nations; for while they have their Ramayuna and Mahavharut, there is not a Hindoo book on history of any particular merit, if we except the fragmentary accounts written by some Cashmerians. To curb this bad habit, and to confine the power of fancy within its proper limits of usefulness is what is urgently required to destroy the unwholesome preponderance so long enjoyed by fiction over fact, and by romance over authentic history.

"This, however, cannot be done without education and strict moral discipline; consequently here is another point to which the attention of every teacher of youth in this country should be especially called."

*Spinal Debility, its Prevention, Pathology, and Cure, in relation to Curvatures, Paralysis, Epilepsy, and various Deformities.* By Edward W. Tuson, F.R.C.S., formerly Surgeon to the Middlesex Hospital. 8vo, London: Davies, 1861, pp. 155.

Mr. Tuson seeks to furnish the Practitioner and Medical Student with the details of some practical observations and facts relative to Spinal Debility, and its consequence. One of the objects the author has in view is "to indicate the necessity of more frequently examining the vertebral column, in cases of spasm, epilepsy, paralysis, and other distressing symptoms, which may often be traced to pressure on the spinal nerves, and relieved by the means narrated."

*Selected Monographs.*—New Sydenham Society, 1861.

This volume will be warmly welcomed by the members of the New Sydenham Society. The monographs consist of Dr. Czermak's treatise on the Laryngoscope, translated from the French edition by Dr. G. D. Gibb; Dr. Dusch's Essay on Thrombosis of the cerebral sinuses, translated by Dr. George Whitley; an elaborate and important report, with observations, of a case of atrophy of the left hemisphere of the brain, with co-existent atrophy of the right side of the body, by Prof. Schroeder van der Kolk, translated by Dr. W. Daniel Moore; a paper by Prof. Rodicke, on the importance and value of arithmetical means with especial reference to recent physiological researches on the determination of the influence of certain agencies upon the metamorphosis of tissue, with rules for accurately estimating the same (a subject new to the English reader, and which the Council have shown great judgment in selecting), translated by Dr. Francis T. Bond; and an essay on the use of cold in surgery by Dr. Esmarch, professor of surgery in the University of Kiel, translated by Dr. Edmund Montgomery.

*A Handbook of the Practice of Forensic Medicine based upon Personal Experience.* By JOHANN LUDWIG CASPER, M.D., Professor of Forensic Medicine in the University of Berlin. Vol. I. Translated from the third edition of the original, by Geo. W. Balfour, M.D.

This is an able translation, revised by the author, of perhaps the most popular German work on Forensic Medicine. The profession are greatly indebted, alike to the New Sydenham Society and Dr. Balfour, for this important and valuable addition to our medical literature. We shall postpone, however, any detailed examination of this translation of Dr. Casper's work until complete.

*The Diseases of the Prostate, their Pathology and Treatment.* By HENRY THOMPSON, F.R.C.S. 8vo, London: Churchill, 1861, pp. 364.

This work comprises the second edition of Mr. Thompson's work, the "Enlarged Prostate," and a dissertation "On the Healthy and Morbid Anatomy of the Prostate Gland," to which the Jacksonian prize for the year 1860 was awarded by the Royal College of Surgeons. Considerable additions have thus been made to the original volume, particularly in what relates to the general, and minute, and morbid anatomy of the prostate, and the subject of Treatment. The sections devoted to morbid anatomy are unrivalled in the extent of the data on which they are based. Several new illustrations have also been added to the work, which will long remain the text-book of English surgeons on the subject.

*A Guide to the Treatment of Diseases of the Skin, with Suggestions for their Prevention.* For the Use of the Student and General Practitioner. Illustrated by Cases. By THOMAS HUNT, F.R.C.S., Surgeon to the Western Dispensary for Diseases of the Skin. Small 8vo. London: Richards, 1861. pp. 256.

This admirable (we might almost say indispensable) little work comes to us, in its fifth edition, weighted with the additional experience of the author, and enriched with an excellent, and most temperate, chapter on the Turkish Bath. We commend the latter, and this edition of Mr. Hunt's work generally, to the attention of our readers.

*On the Parasitic Affections of the Skin.* By T. M'CALL ANDERSON, M.D., Fellow of the Faculty of Physicians and Surgeons, Physician to the Dispensary for Skin Diseases, Glasgow, &c. 8vo. London: Churchill, 1861. pp. 152.

This work is an extension of several articles which originally appeared in the *Medical Times and Gazette.* Dr. Anderson has done well to collect these articles and publish them in a separate form, and his work will be most acceptable to the practitioner and student. The work treats of Favus (due to the presence of the *Achorion Schönleini*), Tinea tonsurans (due to the presence of *Tricophyton*), Alopecia areata (due to the presence of *Microsposon Audouini*), Pityriasis versicolor (due to *Microsposon Furfur*), Phthiriasis (due to *Pediculi*), and Scabies (due to the *Acarus Scabiei*). The different chapters are carefully illustrated.

*Transactions of the Medical Society of London.* Vol. i., pts. 2 and 3, 8vo. Richards, 1861.

The second part of these *Transactions* is entirely devoted to Dr. B. W. Richardson's Lettsomian Lectures "On Certain of the Phenomena of Life." The first of these important Lectures treats of the process of oxygenation in animal bodies; the second, on points relating to the calorification of animal bodies; and the third, on reanimation after certain forms of death. The value and suggestiveness of these Lectures are already well known and appreciated by the profession.

The third part of the *Transactions* contains papers on the following subjects:—"Some Suggestions for an Improved Practice in Strangulated Hernia," by Mr. Bryant; "On Intestinal Obstruction by the Solitary Band," by Mr. Gay; "On Congenital Phymosis considered as a frequent Cause of Irritation of the Genito-Urinary Organs of Young Children," by Mr. Price; and "On Progressive Muscular Atrophy," by Dr. E. Symes Thompson.

It is gratifying to find that the Medical Society has fairly adventured upon the experiment of publishing its Transactions; but we may be permitted to wonder that so long a period has elapsed before the need of such a course seems to have dawned upon the Society.

*Logic for the Million; a familiar Exposition of the Art of Reasoning, with an Appendix on the Philosophy of Language.* By J. W. Gilbart, F.R.S. *Sixth Edition.* Small 8vo. London: Longman, 1860. pp. 390.

The fact of this being the *sixth* edition of this charming work, is a sufficient indication of its worth and of the esteem in which it is justly held. Mr. Gilbart's method of illustrating his propositions by actual and not fictitious examples, gives peculiar value to his treatise as a work of instruction, and imparts to his pages a perennial freshness (if we may so speak).

*Observations and Experiments on the Carcinus Mænas.* By W. Carmichael McIntosh, M.D. Prize Thesis, 1860. 8vo. London: Williams and Norgate. 1861. pp. 64.

A careful and interesting series of observations on the nervous system of the common shore crab, together with experiments upon the influence exercised on the animal by different vapours and poisons. But we question whether many of the latter were either called for, or could lead to any results whatever justifying their adoption. What, for example, was sought for, or what intended, by pouring collodion on the maxillæ and into the mouth of a crab?

*Hints on the Preservation of Health in Armies.* For the use of Volunteer Officers and Soldiers. By John Ordronaux, M.D., Professor of Medical Jurisprudence in Columbia College, New York. New York, 1861. pp. 142.

This is a useful little manual, or, as its author says, "horn-book," on military hygiene, specially adapted for volunteer officers and soldiers. The idea is a good one, and might be advantageously followed here.

We have also lying before us, Dr. W. Lauder Lindsay's elaborate *Report of James Murray's Royal Asylum for Lunatics, near Perth* (the 34th Annual Report of the Asylum); Reports of the *Sussex County Lunatic Asylum, Hayward's Heath,* and of the *Eastern Lunatic Asylum, Kentucky:* also, a reprint of Dr. Lockhart Robinson's *Case of Homicidal Mania without Disorder of Intellect,* from the *Journal of Mental Science;* and of Mr. E. C. Hulme's beautifully illustrated "Case of Irideremia Totalis," from the *Medico-Chirurgical Transactions.*

